# Repurposing New Use for Old Drug Chloroquine against Metabolic Syndrome: A Review on Animal and Human Evidence

**DOI:** 10.7150/ijms.58147

**Published:** 2021-05-13

**Authors:** Sok Kuan Wong

**Affiliations:** Department of Pharmacology, Faculty of Medicine, Universiti Kebangsaan Malaysia, Jalan Yaacob Latif, Bandar Tun Razak, 56000 Cheras, Kuala Lumpur, Malaysia.

**Keywords:** diabetes, dyslipidaemia, hypertension, immunomodulation, inflammation, obesity

## Abstract

Chloroquine (CQ) and hydroxychloroquine (HCQ) are traditional anti-malarial drugs that have been repurposed for new therapeutic uses in many diseases due to their simple usage and cost-effectiveness. The pleiotropic effects of CQ and HCQ in regulating blood pressure, glucose homeostasis, lipid, and carbohydrate metabolism have been previously described* in vivo* and in humans, thus suggesting their role in metabolic syndrome (MetS) prevention. The anti-hyperglycaemic, anti-hyperlipidaemic, cardioprotective, anti-hypertensive, and anti-obesity effects of CQ and HCQ might be elicited through reduction of inflammatory response and oxidative stress, improvement of endothelial function, activation of insulin signalling pathway, inhibition of lipogenesis and autophagy, as well as regulation of adipokines and apoptosis. In conclusion, the current state of knowledge supported the repurposing of CQ and HCQ usage in the management of MetS.

## Introduction

Chloroquine (CQ) and hydroxychloroquine (HCQ), first approved for medical use in 1949 and 1955 respectively, have been widely recognised for its effectiveness in the treatment of malaria and autoimmune diseases such as rheumatoid arthritis and systemic lupus erythematosus (SLE). They exert anti-plasmodial effect against *Plasmodium* parasites by inhibiting the chemical polymerisation of toxic heme released during proteolysis of haemoglobin in the parasites' digestive vacuole 1. Autoimmune diseases are caused by dysfunction of immune system that attacks and damages healthy tissues instead of foreign invaders. Both CQ and HCQ possess anti-inflammatory and immunomodulatory actions which account for their immunosuppressive capability during autoimmune disorders, mainly through (a) the inhibition of lysosomal enzyme activity that blocks the antigen presentation and (b) the suppression of Toll-like receptor (TLR) signalling pathway to reduce the synthesis of pro-inflammatory cytokines 2. Research advancement also revealed the anti-viral properties of CQ and HCQ to fight against rabies 3, polio 4, Chikungunya 5, dengue 6,7, Zika 8, herpes simplex 9, human immunodeficiency 10, hepatitis C 11, and influenza 12 viruses. Most recently, CQ and HCQ have received much attention due to the purported efficacy as promising candidates to treat the novel coronavirus (COVID-19) 13. Apart from that, these anti-malarial drugs have been also reported to have pleiotropic effects in multifarious non-rheumatoid conditions such as infectious diseases, coagulopathies, bone disorders, malignancies, atherosclerosis, diabetes, disordered lipid metabolism, and hypertension 14,15. The latter three conditions are closely associated with metabolic syndrome (MetS).

Metabolic syndrome is a complex medical condition with co-existence of three out of five abnormalities that predispose an individual to a higher risk of cardiovascular disease and stroke 16. The risk factors for MetS include increased waist circumference, blood pressure (BP), fasting blood glucose (FBG), triglycerides (TG) and reduced high-density lipoprotein cholesterol (HDL-C). A close relationship between MetS as well as malaria, rheumatic disorders, and virus infections has been previously elucidated whereby the presence of obesity, diabetes, hypertension, and/or dyslipidaemia can aggravate these diseases. There were more death cases and higher severity of malaria in diabetic patients than non-diabetics 17. A cross-sectional study in North India reported a higher prevalence of MetS in human immunodeficiency virus-positive patients 18. Another cross-sectional study assessed the prevalence of MetS in adult patients with SLE in Argentina. The findings concluded a significantly higher prevalence of MetS and frequency of arterial hypertension in the SLE patients than the control subjects. The use of HCQ was negatively associated with MetS (odds ratio: 0.13, 95% confidence interval: 0.03-0.68), indicating that HCQ was protective against the presence of MetS 19. Hence, the repurposing of CQ and HCQ as a treatment for MetS remains clinically significant.

This review focused on summarising the potential role of CQ and HCQ in preventing MetS and its associated medical conditions. The underlying molecular mechanisms governing the positive effects of CQ and HCQ are also highlighted. We hope to provide information for healthcare providers and researchers about the possibility of expanding the clinical applicability for CQ and HCQ.

## The anti-hyperglycaemic action of chloroquine

### Evidence from animal studies

*In vivo* studies investigating the hypoglycaemic effects of CQ or HCQ have been conducted since several decades ago using diabetic and healthy animals (Table 1). Streptozotocin (STZ) is an anti-neoplastic agent that damages the insulin-producing beta cells of the pancreas, thus it is commonly used in the induction of type 1 diabetes mellitus in animals 20. In one of the earlier studies, Asamoah and colleagues pre-treated female Sprague-Dawley rats with CQ (20 mg/kg) twice a week for 12 weeks via intramuscular (i.m.) injection and followed by induction of diabetes using 50 mg/kg STZ through intravenous (i.v.) injection. Results from this study indicated that CQ lowered FBG, glycated haemoglobin (HbA1c), glycated plasma protein and increased plasma immunoreactive insulin in the diabetic rats 21. Using male Sprague-Dawley rats, Emami and co-authors intraperitoneally (i.p.) injected the animals with STZ to induce diabetes and orally supplemented them with HCQ (80, 120 or 160 mg/kg/day) for 10 days. Treatment with HCQ showed dose-dependent effects in lowering serum glucose and increasing serum insulin 22. In another study, the STZ-induced diabetic rats were orally treated with 200 mg/kg/week HCQ for four weeks. Reductions of FBG, oral glucose tolerance test (OGTT) as well as increases of insulin level and β-cell function were observed in the animals. Histologically, the islets of Langerhans showed preserved structure, intact cellular component, minimal hyaline deposition, and absence of inflammatory cells. Immunohistochemical results revealed that the islets of Langerhans of HCQ-treated diabetic rats exhibited more intense insulin expressing cells and less predominant glucagon expressing cells 23. However, a study by Yuan et al. showed opposite outcome whereby administration of CQ (60 mg/kg/day, i.p.) for 14 days did not have any effect on blood glucose level in STZ-induced male mice 24. The negligible effects observed in this study might be due to the difference in treatment dose, duration and mode of administration. Alloxan is a hydrophilic glucose analogue with preferential toxicity towards pancreatic beta cells, which is also used to induce insulin-dependent diabetes mellitus 25. Pareek et al. utilized a diabetic rat model induced by alloxan monohydrate to investigate the effect of HCQ on blood glucose. Similar to most of the studies using STZ-induced diabetic animals, they also reported a fall in glucose level in diabetic mice following HCQ administration 26.

Apart from that, researchers have investigated the anti-hyperglycaemic actions of CQ and HCQ in diet-induced animal models. Halaby et al. induced insulin resistance in male Wistar rats using a high-fat diet containing 35% lard. After three months of induction, the animals were intraperitoneally administered with 3.5 mg/kg CQ twice per week for one month. As compared to the negative controls, the CQ-treated animals had higher glucose uptake in L6 muscle cells 27. Subsequently, another group of researchers pointed out that oral treatment with HCQ (6.5 mg/kg/day) for 12 weeks lowered FBG, OGTT, insulin, homeostatic model assessment for insulin resistance (HOMA-IR), and raised β-cell function in rats fed with high-fat diet containing 46% fat, 20.3% protein, and 24% carbohydrate. Histological findings from this study also showed that the structure of islets of Langerhans was preserved, cellular component was intact as well as hyaline deposition and inflammatory cell infiltration were absent in the CQ-treated animals. Regression of islets of Langerhans expansion, mild reaction of insulin-secreting cells and more abundant glucagon expressing cells were also seen in the high-fat diet-fed animals with HCQ treatment 28. More recently, Qiao and co-researchers found that pre-treatment of HCQ (40 mg/kg/day) via intraperitoneal injection to male C57BL/6J mice before subjected the animals with high-fat diet significantly reduced intraperitoneal glucose tolerance test (IPGTT), insulin, insulin tolerance test (ITT), HOMA-IR as well as increased homeostatic model assessment for insulin sensitivity (HOMA-IS) and glucose uptake in HepG2 cells 29.

In a genetically diabetic (db/db) mouse model, oral treatment with 50 mg/kg HCQ did not cause a significant change in glucose level as compared to the control group after one week. However, the level of insulin in the HCQ-treated mice was increased 30. The diabetic characteristics of db/db mouse model are derived from single autosomal recessive mutation in leptin receptor gene on chromosome 4, whereby the defective leptin receptor results in excessive extracellular leptin but lack of intracellular leptin 31. The impairment of leptin signalling perturbs the physiological processes of glucose utilisation, energy expenditure and insulin sensitivity 32. Thus, it has been postulated that HCQ might be inefficient to alleviate insulin resistance caused by attenuation of leptin signalling in the leptin receptor-deficient mice. In normal animals, studies found both positive and negligible effects of CQ and HCQ on parameters related to hyperglycaemia. Intramuscular injection of CQ (20 mg/kg) thrice weekly into female normal Sprague-Dawley rats resulted in the decreases in FBG, HbA1c and increase in insulin level 21. In contrast, a study by Emami et al. described that oral HCQ (160 mg/kg) treatment for 10 days did not influence FBG and IPGTT in healthy rats 22. The reduction of glucose level might be minimal owing to the normal glycaemic status in healthy animals. Findings from these studies suggested that CQ and HCQ might exert lesser glycaemic control in genetically diabetic animals and normal healthy animals.

Taken together, most of the studies indicated the positive effects of CQ and HCQ in hindering of hyperglycaemic condition in animals except for few studies. The heterogeneous outcomes might be attributed to the variation in treatment dose, duration, route of administration and type of animal model.

### Evidence from human studies

Numerous human studies have been performed to investigate the relationship between CQ or HCQ use and risk of diabetes as well as the effects of CQ or HCQ in preventing hyperglycaemia. These studies were conducted in patients displaying various types of medical conditions such as type 2 diabetes mellitus (T2DM), obesity, MetS, dyslipidaemia, rheumatoid arthritis and SLE (Table 2).

In a retrospective cohort consisting of 1127 adults aged 18 years and above with newly diagnosed rheumatoid arthritis and no diabetes, Bili and colleagues classified the patients into two subgroups (ever users and never users of HCQ) to examine the relationship between HCQ use and incidence of diabetes. The hazard ratio (HR) for diabetes incident among HCQ users was 0.29 [95% confidence interval (CI) 0.09-0.95], reiterating the potential of HCQ in reducing the risk of diabetes in rheumatoid arthritis patients 33. A nationwide population-based cohort study was implemented to study the association between HCQ use and risk of diabetes mellitus in SLE patients. This study enrolled a total of 221 SLE patients with newly diagnosed diabetes mellitus and they were divided into those had taken or had never taken HCQ. The data collected from this study demonstrated that the use of HCQ was associated with a lower risk of diabetes mellitus incident, with the reported HR for diabetes incident among HCQ users was 0.26 [95% CI 0.18-0.37] 34.

In prediabetic patients (n=20; aged 45.9 ± 7.32 years), a randomised double-blinded controlled trial revealed an increase in insulin level and a decrease in OGTT after 12 weeks of HCQ initiation at the dose of 6.5 mg/kg/day 35. In diabetic patients (n=45; aged 61 ± 13 years), there was a significantly greater reduction in HbA1c after 12 months of HCQ treatment 36. Studies conducted by Powrie and co-authors determined the effects of three days oral CQ administration on FBG and fasting plasma insulin among patients with non-insulin-dependent diabetes mellitus (NIDDM) (n=10; aged 43 - 61 years). CQ caused a decrease in FBG and an increase in fasting plasma insulin in these patients 37,38. Pareek et al. carried out a double-blind study to test the efficacy of HCQ as a treatment of hyperglycaemia in the Indian population. Patients with uncontrolled T2DM (n=135; mean age 52.60 ± 8.55 years) receiving 400 mg HCQ daily for 24 weeks exhibited lower HbA1c, FBG and postprandial glucose at week 12 and week 24 as compared to baseline 39. Gerstein et al. designed a randomised trial to assess the glucose-lowering efficacy and responsiveness of HCQ in sulfonylurea-refractory T2DM patients (n=69; mean age: 58 ± 9.6 years). Participants were given 300 mg HCQ daily. The level of HbA1c was decreased and glucose tolerance was improved after 6 months of supplementation 40. More recently, another randomised controlled trial determined the effects of HCQ as an anti-hyperglycaemic agent in T2DM patients failing metformin and sulfonylurea (n=15; aged 18-75 years). It was noted that the HbA1c and FBG levels were significantly reduced throughout the study period of four months 41.

Mercer et al. conducted a longitudinal study to determine the effects of HCQ (6.5 mg/kg/day) treatment for six weeks on insulin resistance, insulin sensitivity and pancreatic β-cell secretion of insulin in obese non-diabetic subjects (n=13; mean age: 49 ± 15 years). The parameters were measured at three time points including week 0 (before HCQ treatment), week 6 (at the end of HCQ treatment) and week 12 (6 weeks after HCQ treatment). There was an increase in insulin sensitivity index (ISI) and a decrease in HOMA-IR after six weeks of HCQ therapy. However, these parameters returned toward baseline at week 12 42. Later on, a randomised, double-blind, parallel-arm trial was done on overweight or obese subjects with one or more markers of insulin resistance aged 18 years and above (n=17) to study the anti-diabetogenic effects of HCQ (400 mg/day). After the intervention, the participants had positive changes in insulin sensitivity and β-cell function as well as negative changes in FBG and HbA1c 43.

Comparable outcomes were observed when a combination of HCQ (200 mg) and atorvastatin (10 mg) was given to the patients with primary dyslipidaemia (n=127; aged 49.21 ± 9.58 years). The levels of HbA1c and FBG were lowered in these patients 44. Recently, a comprehensive study consisting of two clinical trials of CQ was conducted in people with MetS. In the first trial, 25 patients aged 18-60 years were evaluated and intervention of CQ was provided in dose escalation. The MetS subjects were given placebo capsules for 21 days (first phase), 80 mg of CQ weekly for 21 days (second phase), 80 mg of CQ daily for 21 days (third phase) and 250 mg of CQ daily for 21 days (fourth phase). A washout period of five to seven weeks was applied after each phase. Results from this trial showed significant suppression of hepatic glucose production, decrease in fasting glucose and increase in hepatic insulin sensitivity 45. In the second trial, 107 patients were included and randomly assigned to placebo (n=51) and 80 mg/day CQ (n=56) for a year. However, the findings showed no effect of CQ in OGTT, HOMA-IR and ISI between the two intervention arms. Their study suggested that long-term treatment with a lower dose of CQ might not be clinically useful for glucose level control 45.

Both CQ and HCQ are prescription for the treatment of SLE and rheumatoid arthritis, thus researchers measured the glycaemic change in these patients after receiving CQ or HCQ. A cross-sectional study recruited non-diabetic women with SLE (n=149) or rheumatoid arthritis (n=177). Among these subjects, 48% of SLE patients and 18% of rheumatoid arthritis patients took HCQ medication at the dose of 400 mg and 200 mg, respectively. The data derived from this study showed that serum glucose was lower in HCQ user than non-users in both SLE and rheumatoid arthritis patients. Lower HOMA-IR was also seen in rheumatoid arthritis patients receiving HCQ 46. In Japanese population, it was also found that patients with SLE or rheumatoid arthritis (n=26; aged 46 ± 16 years) prescribed with HCQ with mean daily dose of 284.6 ± 67.5 mg had lower HbA1c after five and six months of HCQ 47. On the contrary, distinct findings were obtained from a randomised, blinded crossover trial. In this study, patients with rheumatoid arthritis without diabetes (n=23; aged 56 ± 11.4 years) were randomised to two groups. One group was allocated to receive 8 weeks of 6.5 mg/kg/day HCQ followed by 8 weeks of placebo whereas another group was assigned to placebo followed by HCQ for the same duration. No statistically significance was observed in ISI and HOMA-IR during HCQ administration as compared to placebo. The authors postulated that a longer treatment duration might allow an apparent anti-hyperglycaemic effect of HCQ in rheumatoid arthritis patients. Besides, observable effect of HCQ in improving insulin and glucose metabolism was not detected in this study as the rheumatoid arthritis patients had normal glycaemic status 48.

In line with *in vivo* studies, most of the human studies indicated that CQ and HCQ users potentially had a lower risk of diabetes, improved insulin sensitivity and glucose tolerance, increased β-cell function, and reduced glucose production in the liver. However, long-term treatment with a lower dose of CQ seemed to have no effect in glycaemic control.

## The anti-hyperlipidaemic action of chloroquine

### Evidence from animal studies

Several preclinical experiments have investigated the anti-hyperlipidaemic actions of CQ and HCQ (Table 3). Favourable effects of HCQ on lipid profile was reported in STZ-induced diabetic rats. Four weeks oral supplementation of 200 mg/kg/day HCQ reduced the levels of total cholesterol (TC), TG, free fatty acid (FFA), low-density lipoprotein cholesterol (LDL-C) and increased HDL-C in the serum of diabetic animals 23. In high-fat diet-fed mice, HCQ (40 mg/kg/day, i.p.) regulated lipid metabolism by reducing TG, TC, FFA and LDL-C in serum in relative to the mice fed with standard chow diet after 17 weeks. The increased amount of TG and TC in liver of high-fat diet-fed mice was also prevented by treatment with HCQ 29. In another study using animals fed with a low protein diet, it was worthy to note that serum TC and TG were lowered after eight weeks of oral CQ (5 mg/kg) administration 49. In contrast with these findings, supplementation of 50 mg/kg HCQ daily for seven days failed to lower the levels of TG, TC and FFA levels in male db/db mice 30. In short, evidence from animal studies suggested that CQ and HCQ might be promising agents in improving lipid profile in animals, but a short duration of treatment may be insufficient to induce these changes.

### Evidence from human studies

The anti-hyperlipidaemic actions of CQ and HCQ have been widely explored in various study populations displaying MetS-associated medical conditions and autoimmune diseases (Table 4). Treatment with CQ and HCQ at the dose between 250-400 mg decreased TC, TG, LDL-C, apolipoprotein B (apoB) and the ratio of apoB to apolipoprotein A-1 (apoA-1) but did not affect non-esterified fatty acids (NEFA) and HDL-C levels in T2DM patients 37,39. A study by Gerstein et al. delineated that HCQ (300 mg/day) reduced LDL-C in T2DM patients showing poor glycaemic control on maximal doses of sulfonylurea after six months of treatment 40. Another recent randomised trial also found that four months supplementation of HCQ at 400 mg lowered TC and non-HDL-C in patients with T2DM who are refractory to sulfonylureas 41. In patients with primary dyslipidaemia receiving a 24-week course of HCQ (200 mg) and atorvastatin (10 mg) in combination, reductions of TC, TG, LDL-C and non-HDL-C were detected 44. McGill and co-researchers conducted two clinical trials of CQ in people with MetS. The first trial indicated the lowering of TC, non-HDL-C and LDL-C after intervention with dose escalation of CQ from 80 mg/week for three weeks, followed by 80 mg/day for subsequent three weeks and 250 mg/day for another three weeks. Similar observations were observed in a second trial whereby the participants were treated with 80 mg/day for one year 45. However, one studies indicated paradoxical outcome. An open-label longitudinal study indicated no change in the levels of TC, TG, HDL-C and LDL-C in obese and non-diabetic subjects (n=13; aged 24 - 71 years) administered with 6.5 mg/kg/day HCQ for six weeks. The small sample size and short treatment period for HCQ might contribute to such distinct findings 42.

Dyslipidaemia is highly prevalent in patients with active autoimmune diseases, such as SLE 50, rheumatoid arthritis 51 and primary Sjögren's syndrome 52. Inconsistent lipid-reducing effects of CQ and HCQ were reported in patients with SLE. In a cross-sectional study, the use of HCQ was negatively associated with development of dyslipidaemia in patients with SLE [relative risk (RR): 0.18; 95% CI: 0.36-5.09] 53. The lipid-lowering effect of 200 mg/day HCQ was noted in SLE patients, observed by reduced TC, LDL-C and frequency of dyslipidaemia after three months of treatment 54. A double-blind, randomised, placebo-controlled, pilot study by Kavanaugh et al. also revealed the alteration of lipoprotein profile [evidenced by decreased TC, TG, very low-density lipoprotein cholesterol (VLDL-C), non-HDL-C, TC/HDL-C ratio and LDL-C/HDL-C ratio] after treated with daily doses of 400 and 800 mg HCQ for three months in SLE patients 55. Nonetheless, two groups of researchers found that CQ and HCQ intakes did not distinguish serum lipid profile in SLE patients as compared to the non-treated control group. In Brazilian population, there was no difference in TC and HDL-C levels between users and non-users of CQ. The authors suggested that the heterogeneity of clinical outcomes might be due to lipid profile was not adjusted for SLE disease activity 56. In SLE patients of Chinese population, researchers were unable to demonstrate significant changes in fasting serum levels of TG, TC, HDL-C and LDL-C between the subjects receiving and not receiving 200-400 mg HCQ daily. These findings could be probably due to the two groups of patients were with mild or inactive disease whereby they had a relatively low level of lipids even not on HCQ treatment 57.

On the other hand, potent anti-hyperlipidaemic activity was consistently reported in rheumatoid arthritis patients. In a study with small sample size consisting of 23 rheumatoid arthritis patients without diabetes (aged 56 ± 11.4 years), Solomon and friends found that the patients allocated to received HCQ (6.5 mg/kg/day) had lower TC and LDL-C after eight weeks 48. In larger study populations, several groups of researchers described comparable findings whereby HCQ use was associated with improved lipid profile (as shown by lowered TG, TC, LDL-C and elevated HDL-C) in individuals with rheumatoid arthritis 58-60. A retrospective cohort study demonstrated a direct relationship between HCQ use and lower risk of hyperlipidaemia among early rheumatoid arthritis patients (HR: 0.75; 95% CI: 0.58-0.98) 61. The use of a triple therapy consisting of methotrexate, sulfasalazine and HCQ was also associated with improved cholesterol profiles over two-year follow-up in early rheumatoid arthritis patients 62. The levels of TC, LDL-C was reduced but HDL-C was increased after individual HCQ administration and its combination with methotrexate and sulfasalazine 61,62. However, the most likely interaction between methotrexate and CQ is the reduction of methotrexate bioavailability when co-administered with CQ 63. In a study recruiting SLE patients (n=22) and rheumatoid arthritis (n=4) patients prescribing HCQ for more than a month, the lipid profile of these patients improved significantly visualised by the lowering of TG, LDL-C and non-HDL-C 47. In addition, significant decreases in TC, atherogenic index and increase in HDL-C were also noted in female patients with Sjögren's syndrome (n=71; aged 64 ± 11 years) receiving HCQ 64.

To sum up, the hypolipidaemic effects of HCQ were suggested based on the currently available evidence. Short treatment duration may be insufficient to induce improvement in lipid profile. In addition, CQ and HCQ may not be able to affect serum lipid levels significantly in patients with mild or inactive autoimmune diseases.

## The cardioprotective and anti-hypertensive actions of chloroquine

Several studies were conducted by investigators to evaluate the cardioprotective and hypotensive effects of CQ and HCQ in animals (Table 5) and humans (Table 6). Yuan et al. found an improvement of diastolic cardiac function in STZ-induced diabetic mice after intraperitoneally administered with 60 mg/kg CQ for 14 days 24. A single monocrotaline (60 mg/kg, s.c.) injection was exposed to adult male Sprague-Dawley rats to induce pulmonary arterial hypertension. The rats were then injected (i.p.) with CQ (20 or 50 mg/kg) or HCQ (50 mg/kg) for 20 days. Lower right ventricular systolic pressure (RVSP), higher cardiac output and contractility were detected in animals after the administration 65. Recently, two studies were done by the same group of researchers to evaluate the anti-hypertensive effect of CQ using adult and young spontaneous hypertensive rats, a genetic animal model with essential hypertension. Therapeutic CQ at the dose of 40 mg/kg/day (i.p.) for 21 days significantly reduced systolic blood pressure (SBP) in spontaneous hypertensive rats of two different ages 66,67. In humans, McGill et al. designed a trial to administer CQ dose escalations (80 mg/week for three weeks followed by 80 mg/day for three weeks and 250 mg/day for three weeks) in MetS patients. The levels of SBP, diastolic blood pressure (DBP) and mean arterial pressure (MAP) were unaffected at the end of study as compared to baseline. However, CQ treatment at 80 mg/day over a year lowered DBP and MAP in MetS subjects 45. In another study, Baker et al. reported a decline in SBP and DBP of patients with prevalent rheumatoid arthritis after initiation of HCQ (dose not mentioned) for 6 months 68. These human studies indicated that long-term administration of CQ and HCQ potentially exhibited anti-hypertensive effects in humans. Although there were not many studies investigated the cardioprotective and anti-hypertensive role of CQ and HCQ, but the findings demonstrated positive outcomes that require further clarification in other animal models and human populations.

## The anti-obesity action of chloroquine

In animals, two studies reported positive effect whereas one study reported negligible effect on body weight and fat mass reduction (Table 7). Obesity was induced in C57BL/6 mice using a high-fat diet and pre-treated with HCQ (40 mg/kg/day, i.p.) for 17 weeks. The treatment significantly reduced body weight gain and fat mass in these mice 29. In line with this study, the findings were supported by McCarthy et al. whereby body weight was reduced in adult spontaneous hypertensive rats treated with CQ (40 mg/kg/day, i.p.) for 21 days 67. Nonetheless, a higher dose of CQ (60 mg/kg/day) provided intraperitoneally to the STZ-induced diabetic mice for 14 days did not change body weight in relative to those treated with vehicle 24. The reduction of body weight was not seen in this study which might be due to the shorter treatment period of CQ. There was only one human study reporting the effects of HCQ (600 mg/day) in combination with doxycycline (100 mg, twice a day) on body weight in healthy individuals and Q fever endocarditis patients (Table 8). The body weight of the subjects increased after one-year treatment. The authors suggested that abnormal weight gain was the side effect of long-term HCQ and doxycycline treatment 69. However, it is difficult to justify whether the adverse effect was derived from HCQ or doxycycline when they were given as a combination therapy. In short, there was only a paucity of scientific studies evaluating the effects of CQ and HCQ on body weight. The measurement of waist circumference should be performed to claim the anti-obesity effect of CQ and HCQ as increased waist circumference is one of the criteria for MetS. More studies are also warranted to validate whether weight gain is the consequence for long-term administration of CQ and HCQ.

## The underlying mechanisms of chloroquine for the prevention of MetS-associated medical conditions

The underlying molecular mechanisms of CQ and HCQ in preventing MetS-associated abnormalities have been uncovered by investigators in recent years. Treatment with CQ and HCQ regulated inflammatory response, oxidative stress, endothelial function, insulin signalling, lipogenesis, autophagy, adipokines, and apoptosis during MetS.

Both CQ and HCQ are commonly used as non-steroidal anti-inflammatory drugs to treat autoimmune diseases, suggesting their potent anti-inflammatory property. Interestingly, chronic low-grade inflammation is the hallmark in the pathogenesis of MetS and its individual medical conditions. Previous animal and human studies have explored the anti-inflammatory and immunomodulatory roles of CQ and HCQ in MetS-related conditions. Abdel-Hamid & El-Firgany found reductions in pro-inflammatory cytokines [interleukin-1 beta (IL-1β), interleukin-6 (IL-6), tumour necrosis factor-alpha (TNF-α), transforming growth factor-beta 1 (TGF-β1) and monocyte chemoattractant protein-1 (MCP-1)] and no change in anti-inflammatory cytokine [interleukin-10 (IL-10)] in STZ-induced diabetic rats after four weeks of oral HCQ administration 23. In high-fat diet-induced obese mice, HCQ decreased the hepatic levels of macrophage-specific genes [cluster of differentiation 68 (CD68) and arginase 1 (Arg1)] and pro-inflammatory genes (IL-1β, TNF-α and MCP-1). Serum levels of inflammatory mediators were also lowered in the HCQ-treated obese mice 29. Treatment of CQ in frequency and dose escalations from 80 mg/week to 250 mg/day for nine weeks helped in reducing the production of TNF-α but did not affect C-reactive protein (CRP) level in patients with MetS 45. The combination of HCQ (200 mg daily) and atorvastatin (10 mg daily) successfully lowered high sensitivity C-reactive protein (hs-CRP) among Indian patients with dyslipidaemia 44. However, a study by Mercer et al. was not in accordance with findings from other groups. They found no significant difference in the levels of CRP and IL-6 in the obese non-diabetic subjects after 6 and 12 weeks of HCQ treatment as compared to their baseline readings. The distinct outcomes might be attributed to the small sample size in this study 42.

The mechanism of action governing the anti-inflammatory property of CQ and HCQ might be mediated through the myeloid differentiation primary response 88 (MyD88)-dependent signalling (also known as the TLR signalling). This signalling pathway is an innate immune system that recognises and responds to pathogen-associated molecular patterns (PAMPs) and damage-associated molecular patterns (DAMPs) to coordinate inflammation during MetS 70. McCarthy et al. investigated the effects of CQ on TLR signalling pathway in young and adult spontaneous hypertensive rats. The expressions of MyD88 and tumour necrosis factor receptor-associated factor 6 (TRAF6) were downregulated by CQ, which eventually decreased phosphorylation of nuclear factor-kappa B (NF-κB), prevented recruitment and infiltration of immune cells into the vasculature as well as lowered SBP. These observations were found in young but not in adult spontaneous hypertensive rats. The authors suggested that the downstream TLR-signalling proteins might be more sensitive to CQ treatment in the young animals, therefore a higher dose of CQ might be needed to exert immunomodulatory effects in the adult animals 66.

High-density lipoprotein (HDL) exerts potent protective effects on cardiovascular system. There are multiple heterogeneous protein components physically associated with HDL such as paraoxonase 1 (PON-1), apoA-1, lecithin cholesterol acyltransferase (LCAT) and platelet-activating factor acetylhydrolase (PAF-AH) 71. HDL acts as an anti-inflammatory particle under physiological condition, but it becomes non-protective and pro-inflammatory in the state of active or chronic inflammation mainly attributed to the changes in the level and function of its associated proteins. PON-1 can bind to enzyme myeloperoxidase (MPO), inhibiting the onset of lipid peroxidation. ApoA-1 helps to solubilise lipid component, reverse cholesterol transport and promote cholesterol efflux from tissue to the liver for excretion 72. Besides, apoA-1 also acts as a co-factor for LCAT, an enzyme that functions to catalyse the esterification of free cholesterol to cholesteryl ester. The effects of HCQ on HDL has been investigated. HCQ combined with methotrexate and sulfasalazine increased PON-1 activity and apoA-1 but decreased HDL inflammatory index and MPO with concomitant improvement of lipid profile in the patients with early rheumatoid arthritis 73. However, it was rather challenging to justify the observed positive effects were derived from HCQ as the intervention was given as combined therapy. In another study, Tam et al. reported that HCQ had a negligible effect on apolipoproteins in Chinese patients with mild or inactive SLE 57. The discrepancy observed in this study might be due to the level of apolipoprotein did not change significantly in mild or inactive conditions of SLE. In addition, the duration of HCQ treatment was not mentioned in this study.

Endothelial dysfunction has been implicated in the pathogenesis of T2DM, insulin resistance, hypertension, hyperlipidaemia, and atherosclerosis, thus it is closely associated with MetS. A reduction in nitric oxide (NO) bioavailability resulted from decreased NO production and/or increased reactive oxygen species (ROS) is involved in the pathophysiology of endothelial dysfunction. In another word, the induction of oxidative stress is also the precursor for endothelial dysfunction. Another mechanism involved in endothelial dysfunction is cyclooxygenase (COX) activity. The stimulation of COX-1 transforms arachidonic acid to endoperoxides, followed by the conversion to various types of prostaglandin depending on their respective synthase. The formation of thromboxane A2 predominates under pathological conditions, which targets thromboxane-prostanoid receptor on smooth muscle cells and activates vasoconstriction 74. Using adult spontaneous hypertensive rats administering with CQ, the hypertensive vascular dysfunction in animals was ameliorated via inhibition of COX enzymes, reduction of ROS generation, improvement of NO bioavailability and reduction of matrix metallopeptidase (MMP) enzymes 67.

CQ and HCQ exert their anti-hyperglycaemic effects by inducing physiological changes in pancreatic beta cells. Previous study has demonstrated that CQ and HCQ are a known inhibitor for Kir6.2 75, an adenosine triphosphate-sensitive potassium channel expressed in many different tissues such as pancreatic beta cells, cardiomyocytes, neurons, and lymphocytes 76-79. The downregulation of Kir6.2 was associated to an increase of insulin secretion in pancreatic beta cell lines 80. Hence, CQ and HCQ potentially increase insulin secretion by interacting with Kir6.2 channels in pancreatic beta cells, resulting in hypoglycaemia.

Apart from that, the insulin signalling pathway plays a central role in maintaining glucose and lipid metabolism. The presence of insulin and insulin growth factor-1 (IGF-1) is recognised by their respective tyrosine kinase receptors [insulin receptor and insulin growth factor-1 receptor (IGF-1R)], leading to activation of phosphatidylinositol 3‑kinase (PI3K) / protein kinase B (Akt) signalling pathway. Insulin receptor substrate-1 (IRS-1) is recruited and activated upon the ligand-receptor interaction, which further activates and phosphorylates PI3K converting phosphatidylinositol 4,5-bisphosphate (PIP_2_) to phosphatidylinositol 3,4,5-trisphosphate (PIP_3_). Subsequently, PIP_3_ recruits phosphoinositide-dependent kinase 1 (PDK1) to phosphorylate Akt. The activation of Akt phosphorylates a series of downstream targets such as and glycogen synthase kinase-3 beta (GSK3β), Forkhead box O (Foxo) and mammalian target of rapamycin (mTOR) that regulate glucose transport, glucose uptake, gluconeogenesis, glycogen synthesis and lipid synthesis 81. The hypoglycaemic action of CQ and HCQ has been reported to mediate through the modulation of insulin signalling pathway. Administration of CQ via injection (i.p.) stimulated glucose uptake and glycogen synthesis in muscle cells of rats adopted with high-fat diet via phosphorylation of Akt (activation) and GSK3β (inhibition). As a result, the glycogen synthase activity was enhanced 27. Wang et al. also verified that supplementation of HCQ in male mice displaying diabetic phenotype resulted in increased insulin level and Akt phosphorylation 30. A recent study by Qiao et al. revealed that the activation of Akt and IRS-1 was detected in liver tissue of male obese mice provided with a high-fat diet and treated with HCQ 29. Findings from these studies summarised the knowledge about the role of CQ and HCQ in activating insulin signalling pathway.

Several molecular mechanisms are implicated in orchestrating lipid metabolism during MetS conditions. Firstly, the activation of peroxisome proliferator-activated receptor-gamma (PPAR-γ) promotes the expression monoacylglycerol-O-acyltransferase (Mgat-1), an enzyme essential for TG synthesis and enhances hepatic lipid accumulation 82. Secondly, the upregulation of sterol regulatory element-binding transcription factor 1 (SREBP1c) and carbohydrate response element-binding protein (ChREBP) enhances the expression of fatty acid synthase (FAS) and acetyl-CoA carboxylase (ACC), facilitating fatty acid biosynthesis and inhibiting fatty acid β-oxidation. Thirdly, the downregulation of carnitine palmitoyltransferase 1 alpha (CPT1α) and carnitine palmitoyltransferase 1 beta (CPT1β) results in the suppression of fatty acid β-oxidation 83. The widely established anti-hyperlipidaemic effects of CQ and HCQ suggested their possible role in modulating lipid metabolism. A recent study reported that HCQ inhibited *de novo* lipogenesis. The expression of PPAR-γ and Mgat-1 (the downstream target), lipogenic genes (SREBP1c and ChREBP), lipid enzymes (FAS and ACC) were downregulated whereas the expression of β-oxidation genes (CPT1α and CPT1β) were upregulated in obese mice fed with high-fat-diet 29.

Adipokines are predominantly secreted by adipose tissue. Dysregulated adipokine levels contribute to the development of various pathological conditions. MetS-related disturbances are positively associated with leptin 84, resistin 85, visfatin 86 but negatively associated with adiponectin 87. On the other hand, lipocalin-2 has both beneficial and detrimental roles in metabolic abnormalities whereby it exerts insulin-sensitising, hepatoprotective, anti- and pro-inflammatory actions 88. In addition, it is evident that these adipokines regulate the expression of intercellular adhesion molecule-1 (ICAM-1), vascular cell adhesion molecule-1 (VCAM-1) and E-selectin, which have direct effects on endothelial function, vascular homeostasis and atherogenesis 89. *In vivo* studies found that HCQ corrected the impaired serum level of adipokines (visualised by raised adiponectin and lipocalin-2 as well as lowered leptin, resistin and visfatin) in the high-fat diet rats after 12 weeks. Along with the changes of adipokine level, significant decreases in serum level of soluble adhesive molecules (E-selectin, ICAM-1 and VCAM-1) were also observed in these animals, suggesting the role of HCQ in relieving endothelial stress 28. A randomised, double-blind, parallel-arm trial pinpointed higher adiponectin level in overweight or obese subjects after HCQ supplementation for 14 weeks but not after placebo supplementation 43. However, a shorter period of CQ treatment (9 weeks) supplemented in dose escalation failed to induce similar changes in leptin and adiponectin levels in patients with MetS 45.

Autophagy is a process of degenerating cells and tissues by clearing old damaged components, misfolded proteins, and intracellular pathogens. Autophagy machinery is initiated with phagophore formation regulated by class III PI3K complex consisting of beclin-1. Next, phagophore membrane is extended and selected cytoplasmic components for degradation tagged by sequestosome-1 (p62) are captured forming an autophagosome. The internalised p62 is degraded, hence higher expression of p62 indicates lower autophagic process. The second step is facilitated by LC3-II protein and various autophagy-related genes (Atg). Lastly, autophagosome is fused with lysosome leading to proteolytic degradation of engulfed molecules 90. Autophagy can be activated under pathological conditions such as type 1 diabetic condition, which is often associated with apoptosis and fibrosis 24. A study by Yuan et al. pointed out that CQ improved cardiac diastolic function by inhibiting autophagy in STZ-induced diabetic mice (evidenced by the decreases in autophagolysosomes, cardiomyocyte apoptosis, cardiac fibrosis, LC3-II/LC3-I protein ratio and increase in p62 expression) 24. Likewise, in a rat model of pulmonary arterial hypertension induced by monocrotaline, the accumulation of p62 protein level was found after CQ injection suggesting that CQ is an inhibitor for autophagy 65.

Apoptosis is a process of programmed cell death mainly regulated by two main branches of signalling pathways, the intrinsic (mitochondrial) and extrinsic (death receptor-mediated) apoptotic pathway, depending on the source of stresses either derived from intracellular or extracellular environment. The intrinsic pathway is triggered by intracellular stressors (such as DNA damage, oxidative stress, and oxygen deprivation), resulting in mitochondrial dysfunction, release of cytochrome c and subsequent activation of caspase-9 and caspase-3. The mitochondrial outer membrane permeabilisation (MOMP) is influenced by several anti-apoptotic genes [B-cell lymphoma 2 (Bcl-2) and B-cell lymphoma-extra large (Bcl-XL)] and pro-apoptotic genes [Bcl-2-associated X protein (Bax) and Bcl-2 homologous antagonist/killer (Bak)]. Meanwhile, the extrinsic pathway is initiated upon the ligation between death ligands and their respective death receptors, leading to the formation of death-inducing signalling complex (DISC), recruitment of Fas-associated death domain (FADD), activation caspase-8 and caspase-3 91. Previous studies by two groups of researchers demonstrated that CQ and HCQ suppressed apoptosis of pancreatic β-cells in diabetic condition but stimulated apoptosis of pulmonary artery smooth muscle cells in hypertensive condition. HCQ induced a significant decrease in cleaved caspase-3 and increase in Bcl-2 in pancreatic islet of Langerhans of diabetic rats as compared to the non-treated diabetic groups 23. On the other hand, Long et al. reported a reduction in the number of Ki67-positive cells and an increase in TUNEL-positive cells in hypertensive rats treated with CQ, indicating the decrease in cell proliferation and increase in apoptosis of pulmonary artery smooth muscle cells 65.

**Figure 1:**
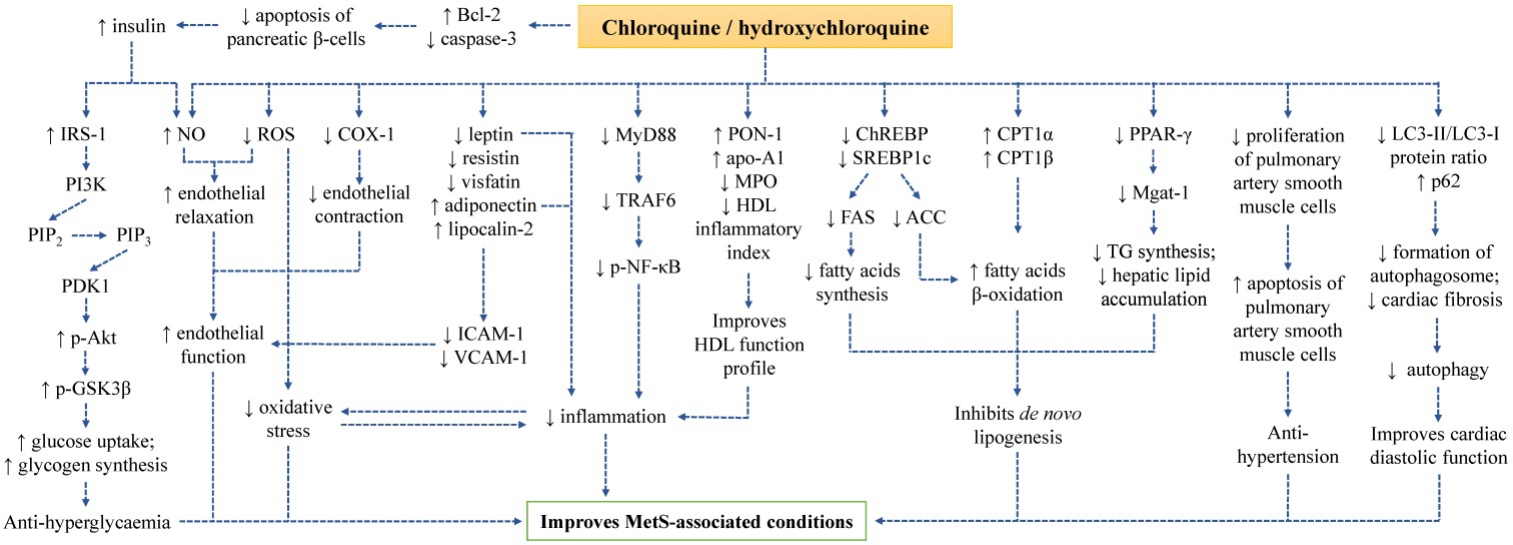
The mechanism of action of CQ and HCQ in improving MetS conditions.

A summary on the mechanism of action of CQ and HCQ in improving MetS-associated conditions has been depicted (Figure 1).

## Perspectives

The intervention dose and duration for CQ and HCQ varies depending on the diseases. A total of 25 mg/kg CQ given in a 3-day course is prescribed to treat malaria caused by *Plasmodium vivax* 92. For rheumatic diseases, the well-tolerated doses for CQ and HCQ are 200-400 mg/day with duration of 24-36 weeks which result in reduced join pain and improved functional response 14,93. The recommended regimen for administration of CQ and HCQ in patients with COVID-19 is 500 mg daily with duration of 7 days 94. Based on the studies included in this review, the dosage of CQ and HCQ commonly used in humans with MetS-associated conditions was 6.5 mg/kg or not exceeding 400 mg/day in adults via oral administration. There was only one study that has tested the anti-hyperlipidaemic effects of 800 mg/day HCQ in patients with SLE 55. The suggested treatment duration for CQ and HCQ ranged from three days to six months, with only few studies with exposure time of approximately two years. On the other hand, the dose for CQ and HCQ treatment in animals ranged from 7 mg/kg/week to 200 mg/kg/day. The human equivalent dose for the highest dose is approximately 2.3 g for a 60 kg adult, which has exceeded the well-tolerated dose in human therefore it was considered as not clinically useful.

The understanding on pharmacokinetics of CQ and HCQ is also a crucial clinical consideration for their possible application in MetS-associated conditions. The oral absorption of CQ and HCQ is rapid and almost complete following ingestion. They possess an estimate bioavailability of 70 - 80% and large volume of distribution in the blood, resulting in a long half-life for the two drugs. Both CQ and HCQ are metabolised by several members of cytochrome P450 family, such as CYP1A2, CYP2C8, CYP2C19, CYP2D6, CYP3A4, and CYP3A5 95. Furthermore, CQ and HCQ can inhibit CYP2D6 activity thus suppressing the metabolism of their own and other drugs metabolised by the enzyme. This suggests the potential clinical interaction with medications that are the predominant substrates of CYP2D6 enzyme.

The proper weight-based daily dosing of CQ and HCQ is crucial to prevent the development of potential adverse events in humans. In a randomised, double-blind, placebo-controlled trial investigating the effects of CQ (500 mg/day, 14-29 days) on breast tumour cellular proliferation in breast cancer patients, the patients receiving CQ developed visual symptoms (such as blurriness and light sensitivity), auditory symptom (tinnitus), muscle weakness, dry mouth, dizziness, fatigue and gastrointestinal upset (such as abdominal cramp, nausea, and diarrhoea) 96. The incidence of gastrointestinal disturbance has been reported as a brand-related side effect 97. Long-term use (more than 6 years) and higher daily administration dose (more than 400 mg) of HCQ have been previously proven as the predictors of retinopathy, an ophthalmologic disorder in humans 98. The occurrence of renal toxicity among anti-malarial drugs is rare yet the main consideration is the use of CQ and HCQ in patients with concomitant renal impairment 99. Hence, patients with renal impairment [estimated glomerular filtration rate (eGFR) ≤ 30 mL/min/1.73 m^2^] are excluded from clinical trials related to CQ and HCQ 100. CQ and HCQ are known to be blockers of the potassium channel. The blockade on potassium channel inhibits potassium current, leading to delayed repolarisation of cardiac myocytes, prolonged cardiac action potential duration and QT interval on electrocardiogram 101. High dose of CQ has been reported to be well-tolerated but it caused QT interval prolongation similar to those receiving standard-dose CQ under clinical conditions 102. In preclinical models, decreased aortic output was observed in rats whereas reduced heart function and heart rate were noted in ventricular cardiomyocytes after CQ treatment 103. Thus, the potential toxicity of CQ and HCQ on the cardiac muscle and large QT syndrome at low doses should not be neglected.

In view of the potential lack of efficiency of CQ and HCQ at short treatment period and the presence of toxicity at higher dose, future studies are warranted to evaluate the optimum treatment dose and duration to ensure the efficacy and safety profile of CQ and HCQ, particularly in subjects with MetS. The combination of lifestyle modification by adopting balanced diet and physical activities with pharmacological treatment is recommended to ensure successful management of MetS-related conditions. The current evidence documented the capability of CQ or HCQ in improving glucose tolerance, insulin sensitivity, lipid profile, cardiac function, reducing BP and weight gain in most of the pre-clinical and human studies. The anti-hyperglycaemic and anti-hyperlipidaemic actions of CQ and HCQ were notable but there was limited literature showing their anti-hypertensive and anti-obesity effects, thus require further investigations to validate the claims. These beneficial outcomes supported the repurposing of CQ and HCQ against MetS initiation and progression despite their current uses as anti-malarial 104, anti-rheumatic 105 and anti-viral drugs 106.

To our knowledge, it is believed that patients with underlying health conditions such as MetS-related abnormalities have an increasingly rapid and severe progression for malaria, autoimmune diseases, virus infections, and vice versa. Obesity, in combination with additional metabolic risk factors such as diabetes, hypertension, and dyslipidaemia were strongly correlated with severe malaria 107. A comprehensive systematic review and meta-analysis reported that SLE patients had higher odds for the risk of high FBG, BP, TG, waist circumference, and low HDL-C 108. Moreover, the prevalence of MetS was higher in rheumatoid arthritis patients than in control subjects 109. With the recent outbreak of COVID-19, patients with comorbidities related to MetS are more likely to develop severe and worsening disease course 110. In this context, the management of MetS should be considered concurrently in the treatment strategies for these diseases. Herein, CQ and HCQ may be a better treatment option for patients with of malaria, autoimmune diseases, and virus infections who are at risk of developing MetS as well as for MetS patients who are at risk of experiencing severe disease state.

## Conclusion

In conclusion, CQ and HCQ have a wide range of biological activities that contribute to their usefulness and effectiveness in the prevention of MetS. The dose and duration of medications need to be paid careful attention. Lower dose and shorter duration may be insufficient to exert beneficial effects whereas higher dose and longer duration may induce adverse symptoms. Besides, the potential adverse reactions of CQ and HCQ to the eyes, ears, muscles, gastrointestinal tract, and heart should be under careful consideration when these drugs are prescribed. Evidence and suggestion from this review awaits further validation from human clinical trials to assess the effects of CQ and HCQ supplementation particularly in MetS subjects to distinctly approve them as a promising agent to improve MetS.

## Figures and Tables

**Table 1:** The anti-hyperglycaemic effects of CQ and HCQ in animal studies

Types of animal	Types of induction	Treatment, dose, route and duration	Research outcomes	Mechanism of action	Reference
Female Sprague-Dawley rats	STZ (50 mg/kg, i.v.)	CQ (20 mg/kg, twice weekly, i.m., 12 weeks)	FBG: ↓, HbA1c: ↓, glycated plasma protein: ↓, insulin: ↑	-	21
	-	CQ (20 mg/kg, thrice weekly, i.m., 20 weeks)	FBG: ↓, HbA1c: ↓, insulin: ↑	-	
Adult male Sprague-Dawley rats	STZ (60 mg/kg, i.p.)	HCQ (80, 120 or 160 mg/kg/day, oral, 10 days)	Glucose: ↓	-	22
	-	HCQ (160 mg/kg/day, oral, 10 days	FBG: ↔	-	
Adult Sprague-Dawley rats	STZ (27.5 mg/kg, i.p.)	HCQ (200 mg/kg/week, oral, 4 weeks)	FBG: ↓, OGTT: ↓, insulin: ↑, β-cell function: ↑	IL-1β: ↓, IL-6: ↓, TNF-α: ↓, TGF-β1: ↓, MCP-1: ↓, IL-10: ↔, caspase-3: ↓, Bcl-2: ↑	23
Male C57BL mice	STZ (60 mg/kg, i.p.)	CQ (60 mg/kg/day, i.p., 14 days)	Glucose: ↔	LC3-II: ↑, p62: ↑, Beclin1: ↔, autophagic vacuoles: ↓	24
Male Wistar rats	Alloxan monohydrate (90 mg/kg, i.p.)	HCQ (200 mg/kg/day, oral, 9 days)	Glucose: ↓	-	26
Male Wistar rats	High-fat diet (35% lard)	CQ (3.5 mg/kg, twice per week, i.p., 1 month)	Glucose uptake in muscle cells: ↑	p-Akt: ↑, p-JNK: ↔, p-GSK3β: ↑, glycogen synthase activity: ↑	27
Adult Sprague-Dawley rats	High-fat diet (46% fat, 20.3% protein, 24% carbohydrate)	HCQ (6.5 mg/kg/day, oral, 12 weeks)	FBG: ↓, OGTT: ↓, insulin: ↓, HOMA-IR: ↓, β-cell function: ↑	E-selectin: ↓, ICAM-1: ↓, VCAM-1: ↓, leptin: ↓, resistin: ↓, visfatin: ↓, adiponectin: ↑, lipocalin-2: ↓	28
Male C57BL/6J mice	High-fat diet (60% fat)	HCQ (40 mg/kg/day, pre-treatment, i.p., 17 weeks)	IPGTT: ↓, insulin: ↓, ITT: ↓, HOMA-IR: ↓, HOMA-IS: ↑, glucose uptake in HepG2 cells: ↑	IL-1β: ↓, IL-6: ↓, TNF-α: ↓, MCP-1: ↓, CD68: ↓, Arg1: ↓, p-Akt: ↑, p-IRS1: ↑	29
Male db/db mice	-	HCQ (50 mg/kg/day, oral, 7 days)	Insulin: ↑, glucose: ↔	IGF-1: ↔, p-Akt: ↑, p-mTOR: ↔, p-S6: ↔	30

Abbreviations: Arg1, arginase 1; Bcl-2, B-cell lymphoma 2; CD, cluster of differentiation; CQ, chloroquine; FBG, fasting blood glucose; HbA1c, glycated haemoglobin; HCQ, hydroxychloroquine; HepG2, human liver cancer cell line; HOMA-IR, homeostatic model assessment for insulin resistance; HOMA-IS, homeostatic model assessment for insulin sensitivity; ICAM-1, intercellular adhesion molecule 1; IGF-1, insulin growth factor 1; IL-1β, interleukin-1 beta; IL-6, interleukin-6; IL-10, interleukin-10; i.m., intramuscular; i.p., intraperitoneal; IPGTT, intraperitoneal glucose tolerance test; ITT, insulin tolerance test; MCP-1, monocyte chemoattractant protein-1; OGTT, oral glucose tolerance test; p-Akt, phosphorylated protein kinase B; p-GSK3β, phosphorylated glycogen synthase kinase-3 beta; p-IRS1, phosphorylated insulin receptor substrate 1; p-JNK, phosphorylated c-Jun N-terminal kinase; p-mTOR, phosphorylated mammalian target of rapamycin; STZ, streptozotocin; TC, total cholesterol; TG, triglyceride; TGF-β1, transforming growth factor-beta 1; TNF-α, tumour necrosis factor-alpha; VCAM-1, vascular cell adhesion molecule 1; ↑, increase/stimulate; ↓, decrease/inhibit; ↔, no change.

**Table 2:** The anti-hyperglycaemic effects of CQ and HCQ in animal studies

Study population	Treatment, dose and duration	Research outcomes	Mechanism of action	Reference
Adults with newly diagnosed rheumatoid arthritis and no diabetes (n=1127, aged ≥18 years)	HCQ (6.5 mg/kg or 400 mg/day)	Risk of diabetes: ↓	-	33
SLE patients with newly diagnosed diabetes mellitus (n=221)	HCQ (cumulative dose ≥ 129 g)	Risk of diabetes: ↓	-	34
Patients with prediabetes (n=20; aged 45.9 ± 7.32 years)	HCQ (6.5 mg/kg/day, 12 weeks)	Insulin: ↑, OGTT: ↓	-	35
Patients with diabetes mellitus (n=45, aged 61 ± 13 years)	HCQ (dose not mentioned, >12 weeks)	HbA1c: ↓	-	36
Patients with T2DM (n=10; aged 43-61 years)	CQ (250 mg, four times daily, 3 days)	FBG: ↓, fasting plasma insulin: ↑	-	37,38
Patients with T2DM (n=135; aged 18-65 years)	HCQ (400 mg/day, 24 weeks)	HbA1c: ↓, FBG: ↓, postprandial glucose: ↓	-	39
Sulfonylurea-refractory patients with poorly controlled T2DM (n=69; aged 35-80 years)	HCQ (300 mg/day, 6 months)	HbA1c: ↓, glucose tolerance: ↑	-	40
Patients with T2DM failing metformin and sulfonylurea (n=15; aged 18-75 years)	HCQ (400 mg/day, 4 months)	HbA1c: ↓, FBG: ↓	-	41
Obese, non-diabetic subjects (n=13; aged 24-71 years)	HCQ (6.5 mg/kg/day, 6 weeks)	ISI: ↑, HOMA-IR: ↓	CRP: ↔, IL-6: ↔	42
Overweight or obese subjects with one or more markers of insulin resistance (n=17; aged 50.1 ± 14.5 years)	HCQ (400 mg/day, oral, 13 ± 1 weeks)	Insulin sensitivity: ↑, β-cell function: ↑, FBG: ↓, HbA1c: ↓	Adiponectin: ↑	43
Patients with primary dyslipidaemia (n=127; aged 49.21 ± 9.58 years)	HCQ (200 mg/day) + atorvastatin (10 mg/day), 24 weeks	HbA1c: ↓, FBG: ↓	hs-CRP: ↓	44
Patients with MetS (n=25; aged 18-60 years)	Placebo (3 weeks) → CQ (80 mg/week, 3 weeks) → CQ (80 mg/day, 3 weeks) → CQ (250 mg/day, 3 weeks)	Hepatic glucose production: ↓, hepatic insulin sensitivity: ↑, FBG: ↓, OGTT: ↔	TNF-α: ↓, CRP: ↔, leptin: ↔, adiponectin: ↔	45
Patients with MetS (n=56; aged 18-70 years)	CQ (80 mg/day, 1 year)	OGTT: ↔, HOMA-IR: ↔, ISI: ↔	p-JNK: ↓	
Women with SLE (n=71; aged 49.8 ± 9.9 years)	HCQ (400 mg, duration not mentioned)	FBG: ↓, HOMA-IR: ↓	-	46
Women with rheumatoid arthritis (n=31; aged 56.5 ± 9.0 years)	HCQ (200 mg, duration not mentioned)	FBG: ↓	-	
Patients with SLE or rheumatoid arthritis(n=26; aged 46 ± 16 years)	HCQ (mean daily dose: 284.6 ± 67.5 mg, 5-6 months)	HbA1c: ↓	-	47
Patients with rheumatoid arthritis without diabetes (n= 23; aged 56 ± 11.4 years)	HCQ (6.5 mg/kg/day, 8 weeks)	ISI: ↔, HOMA-IR: ↔	-	48

Abbreviations: CQ, chloroquine; CRP, C-reactive protein; FBG, fasting blood glucose; HbA1c, glycated haemoglobin; HCQ, hydroxychloroquine; HOMA-IR, homeostatic model assessment for insulin resistance; hs-CRP, high sensitivity C-reactive protein; IL-6, interleukin-6; ISI, insulin sensitivity index; MetS, metabolic syndrome; OGTT, oral glucose tolerance test; p-JNK, phosphorylated c-Jun N-terminal kinase; SLE, systemic lupus erythematosus; TNF-α, tumour necrosis factor-alpha; T2DM, type 2 diabetes mellitus; ↑, increase/stimulate; ↓, decrease/inhibit; ↔, no change.

**Table 3:** The anti-hyperlipidaemic effects of CQ and HCQ in animal studies

Types of animal	Types of induction	Treatment, dose, route and duration	Research outcomes	Mechanism of action	Reference
Adult Sprague-Dawley rats	STZ (27.5 mg/kg, i.p.)	HCQ (200 mg/kg/week, oral, 4 weeks)	TG: ↓, TC: ↓, FFA: ↓, LDL-C: ↓, HDL-C: ↑	IL-1β: ↓, IL-6: ↓, TNF-α: ↓, TGF-β1: ↓, MCP-1: ↓, IL-10: ↔	23
Male C57BL/6J mice	High-fat diet (60% fat)	HCQ (40 mg/kg/day, pre-treatment, i.p., 17 weeks)	Serum TG: ↓, TC: ↓, LDL-C: ↓, FFA: ↓, HDL-C: ↔ Liver TG: ↓, TC: ↓	Inflammation IL-1β: ↓, IL-6: ↓, TNF-α: ↓, MCP-1: ↓, CD68: ↓, Arg1: ↓ Lipid metabolism CPT1α: ↑, CPT1β: ↑, PPAR-γ: ↓, Mgat-1: ↓, SREBP1c: ↓, ChREBP: ↓, ACC: ↓, FAS: ↓	29
Male Wistar rats	Low protein diet	CQ (5 mg/kg, 3 days/week, oral, 8 weeks)	TC: ↓, TG: ↓	-	49
Male db/db mice	-	HCQ (50 mg/kg/day, oral, 7 days)	FFA: ↔, TG: ↔, TC: ↔, TC (heart): ↔	IGF-1: ↔, p-Akt: ↑, p-mTOR: ↔, p-S6: ↔	30

Abbreviations: ACC, acetyl-CoA carboxylase; Arg1, arginase 1; CD, cluster of differentiation; ChREBP, carbohydrate response element binding protein; CPT1α, carnitine palmitoyltransferase 1 alpha; CPT1β, carnitine palmitoyltransferase 1 beta; CQ, chloroquine; FAS; fatty acid synthase; FFA, free fatty acids; HCQ, hydroxychloroquine; HDL-C, high-density lipoprotein cholesterol; IGF-1, insulin growth factor 1; IL-1β, interleukin-1 beta; IL-6, interleukin-6; IL-10, interleukin-10; i.p., intraperitoneal; LDL-C, low-density lipoprotein cholesterol; MCP-1, monocyte chemoattractant protein-1; Mgat-1, monoacylglycerol-O-acyltransferase; p-Akt, phosphorylated protein kinase B; p-mTOR, phosphorylated mammalian target of rapamycin; PPAR-γ, peroxisome proliferator-activated receptor-gamma; SREBP1c, sterol regulatory element-binding transcription factor 1; STZ, streptozotocin; TC, total cholesterol; TG, triglyceride; TGF-β1, transforming growth factor-beta 1; TNF-α, tumour necrosis factor-alpha; ↑, increase/stimulate; ↓, decrease/inhibit; ↔, no change.

**Table 4:** The anti-hyperlipidaemic effects of CQ and HCQ in human studies

Study population	Treatment, dose and duration	Research outcomes	Mechanism of action	Reference
Patients with T2DM (n=10; aged 43-61 years)	CQ (250 mg, four times daily, 3 days)	TC: ↓, LDL-C: ↓, apoB: ↓, apoB/apoA-1 ratio: ↓, NEFA: ↔	-	37
Patients with T2DM (n=135; aged 18-65 years)	HCQ (400 mg/day, 24 weeks)	TC: ↓, TG: ↓, LDL-C: ↓, HDL-C: ↔	-	39
Sulfonylurea-refractory patients with poorly controlled T2DM (n=69; aged 35-80 years)	HCQ (300 mg/day, 6 months)	LDL-C: ↓	-	40
Patients with T2DM failing metformin and sulfonylurea (n=15; aged 18-75 years)	HCQ (400 mg/day, 4 months)	TC: ↓, non-HDL-C: ↓, TG: ↔, LDL-C: ↔, HDL-C: ↔	-	41
Patients with primary dyslipidaemia (n=127; aged 49.21 ± 9.58 years)	HCQ (200 mg/day) + atorvastatin (10 mg/day), 24 weeks	TC: ↓, TG: ↓, LDL-C: ↓, non-HDL-C: ↓, HDL-C: ↔	hs-CRP: ↓	44
Patients with MetS (n=25; aged 18-60 years)	Placebo (3 weeks) → CQ (80 mg/week, 3 weeks) → CQ (80 mg/day, 3 weeks) → CQ (250 mg/day, 3 weeks)	TC: ↓, non-HDL-C: ↓, LDL-C: ↓, TG: ↔, HDL-C: ↔, NEFA: ↔	TNF-α: ↓, CRP: ↔, leptin: ↔, adiponectin: ↔	45
Patients with MetS (n=56; aged 18-70 years)	CQ (80 mg/day, 1 year)	TC; ↓, non-HDL-C: ↓, LDL-C: ↓	p-JNK: ↓	
Obese, non-diabetic subjects (n=13; aged 24-71 years)	HCQ (6.5 mg/kg/day, 6 weeks)	TC: ↔, TG: ↔, HDL-C: ↔, LDL-C: ↔	CRP: ↔, IL-6: ↔	42
Patients with SLE (n=51; aged ≥18 years)	HCQ (dose and duration not mentioned)	Dyslipidaemia: ↓	-	53
Patients with SLE (n=24; aged 37.2 ± 16.0 years)	HCQ (200 mg/day) - 3 months	TC: ↓, LDL-C: ↓, frequency of dyslipidaemia: ↓	-	54
Female patients with SLE (n=17)	HCQ (400 or 800 mg/day) - 3 months	TC: ↓, TG: ↓, VLDL-C: ↓, LDL-C: ↔, HDL-C: ↔, non-HDL-C: ↓, TC/HDL-C ratio: ↓, LDL-C/HDL-C ratio: ↓	-	55
Patients with SLE (n=34; aged 48.7 ± 13.3 years)	HCQ (standard dose)	TC: ↔, HDL-C: ↔	-	56
Chinese patients with mild or inactive SLE (n=44; aged 38.9 ± 7.9 years)	HCQ (244 ± 86 mg/day, duration not mentioned)	TG: ↔, TC: ↔, HDL-C: ↔, LDL-C: ↔	apoA-1: ↔, apoB: ↔, lipoprotein A: ↔	57
Patients with rheumatoid arthritis without diabetes (n= 23; aged 56 ± 11.4 years)	HCQ (6.5 mg/kg/day, 8 weeks)	TC: ↓, LDL-C: ↓, TG: ↔, HDL-C: ↔	-	48
Male and female patients with rheumatoid arthritis (n=150; aged 65.8 ± 10.0 years)	HCQ (dose not mentioned, >3 months)	TG: ↓, TC: ↓, LDL-C: ↓, HDL-C: ↔, HDL-C/LDL-C: ↑, TC/HDL-C: ↓	-	58
Patients with rheumatoid arthritis (n=256; aged 54-72 years)	HCQ (6.5 mg/kg/day, median exposure time: 1.98 years)	TG: ↓, TC: ↓, LDL-C: ↓, HDL-C: ↑, LDL-C/HDL-C: ↓, TC/HDL-C: ↓	-	59
Patients with rheumatoid arthritis (n=254; aged 57.2 ± 11.1 years)	HCQ (dose and duration not mentioned)	TG: ↓, TC: ↓, LDL-C: ↓, HDL-C: ↑, TC/HDL-C: ↓, LDL-C/HDL-C: ↓	-	60
Patients with early rheumatoid arthritis (n=6130; aged 45.45 ± 12.12 years)	HCQ (dose and duration not mentioned)	TC: ↓, LDL-C: ↓, HDL-C: ↑	-	61
Patients with early rheumatoid arthritis (n=119; aged 48.66 ± 11.98 years)	Methotrexate + sulfasalazine + HCQ - 24, 48 and 102 weeks	TC: ↓, LDL-C: ↓, HDL-C: ↑	-	62
Patients with early rheumatoid arthritis (n=103; aged 49.3 ± 12.5 years)	Methotrexate + sulfasalazine + HCQ - 24, 48 and 102 weeks	-	PON-1 activity: ↑, apoA-1: ↑, HDL inflammatory index: ↓, MPO: ↓	73
Patients with SLE or rheumatoid arthritis (n=26; aged 46 ± 16 years)	HCQ (mean daily dose: 284.6 ± 67.5 mg, 5 - 6 months)	TG: ↓, HDL-C: ↔, LDL-C: ↓, non-HDL-C: ↓	-	47
Female patients with Sjögren syndrome (n=71; aged >18 years)	HCQ	TC: ↓, HDL-C: ↑, atherogenic index: ↓	-	64

Abbreviations: apoA-1, apolipoprotein A-1; apoB, apolipoprotein B; CQ, chloroquine; CRP, C-reactive protein; HCQ, hydroxychloroquine; HDL-C, high-density lipoprotein cholesterol; hs-CRP, high sensitivity C-reactive protein; IL-6, interleukin-6; LDL-C, low-density lipoprotein cholesterol; MetS, metabolic syndrome; MPO, myeloperoxidase; NEFA, non-esterified fatty acids; p-JNK, phosphorylated c-Jun N-terminal kinase; PON-1, paraoxonase 1; SBP, systolic blood pressure; SLE, systemic lupus erythematosus; TC, total cholesterol; TG, triglyceride; TNF-α, tumour necrosis factor-alpha; T2DM, type 2 diabetes mellitus; VLDL-C, very low-density lipoprotein cholesterol; ↑, increase/stimulate; ↓, decrease/inhibit; ↔, no change.

**Table 5:** The cardioprotective and anti-hypertensive effects of CQ and HCQ in animal studies

Types of animal	Types of induction	Treatment, dose, route and duration	Research outcomes	Mechanism of action	Reference
Male C57BL mice	STZ (60 mg/kg, i.p.)	CQ (60 mg/kg/day, i.p., 14 days)	Diastolic cardiac function: ↑	LC3-II: ↑, p62: ↑, Beclin1: ↔, autophagic vacuoles: ↓	24
Male Sprague-Dawley rats	Monocrotaline (60 mg/kg)	CQ (20 or 50 mg/kg/day, i.p., 20 days)	RVSP: ↓, cardiac output: ↑, cardiac contractility: ↑	p62: ↑, Ki67: ↓, TUNEL-positive cells: ↑, BMPR-II: ↑	65
		HCQ (50 mg/kg/day, i.p., 20 days)	RVSP: ↓	-	
Young spontaneous hypertensive rats	-	CQ (40 mg/kg/day, i.p., 21 days)	SBP: ↓	MyD88: ↓, TRAF6: ↓, p-NF-κB: ↓, immune cell recruitment and infiltration into the vasculature: ↓	66
Adult spontaneous hypertensive rats	-	CQ (40 mg/kg/day, i.p., 21 days)	-	TLR8: ↑, MyD88: ↑, IRAK: ↑, p-NF-κB: ↑	
Adult spontaneous hypertensive rats	-	CQ (40 mg/kg/day, i.p., 21 days)	SBP: ↓	COX enzymes: ↓, ROS: ↓, nitric oxide: ↑, MMP: ↓	67

Abbreviations: BMPR-II, bone morphogenetic protein receptor type II; COX, cyclooxygenase; CQ, chloroquine; HCQ, hydroxychloroquine; i.p., intraperitoneal; IRAK, IL-1 receptor-associated kinase; MMP, matrix metallopeptidase; MyD88; myeloid differentiation primary response 88; p-NF-κB, phosphorylated nuclear factor-kappa B; ROS, reactive oxygen species; RVSP, right ventricular systolic pressure; STZ, streptozotocin; TLR8, Toll-like receptor 8; TRAF6, tumour necrosis factor receptor-associated factor 6; ↑, increase/stimulate; ↓, decrease/inhibit; ↔, no change.

**Table 6:** The cardioprotective and anti-hypertensive effects of CQ and HCQ in human studies

Study population	Treatment, dose and duration	Research outcomes	Mechanism of action	Reference
Patients with MetS (n=25; aged 18-60 years)	Placebo (3 weeks) → CQ (80 mg/week, 3 weeks) → CQ (80 mg/day, 3 weeks) → CQ (250 mg/day, 3 weeks)	BP: ↔	TNF-α: ↓, CRP: ↔, leptin: ↔, adiponectin: ↔	45
Patients with MetS (n=56; aged 18-70 years)	CQ (80 mg/day, 1 year)	DBP: ↓, MAP: ↓	p-JNK: ↓	
Patients with prevalent rheumatoid arthritis (n=7,147; aged 63 years)	HCQ (dose not mentioned) - 6 months	SBP: ↓, DBP: ↓	-	68

Abbreviations: BP, blood pressure; CQ, chloroquine; CRP, C-reactive protein; DBP, diastolic blood pressure; HCQ, hydroxychloroquine; MAP, mean arterial pressure; MetS, metabolic syndrome; p-JNK, phosphorylated c-Jun N-terminal kinase; SBP, systolic blood pressure; TNF-α, tumour necrosis factor-alpha; ↑, increase/stimulate; ↓, decrease/inhibit; ↔, no change.

**Table 7:** The anti-obesity effects of CQ and HCQ in animal studies

Types of animal	Types of induction	Treatment, dose, route and duration	Research outcomes	Mechanism of action	Reference
Male C57BL/6J mice	High-fat diet (60% fat)	HCQ (40 mg/kg/day, pre-treatment, i.p., 17 weeks)	Weight gain: ↓, fat mass: ↓	Inflammation IL-1β: ↓, IL-6: ↓, TNF-α: ↓, MCP-1: ↓, CD68: ↓, Arg1: ↓ Lipid metabolism CPT1α: ↑, CPT1β: ↑, PPAR-γ: ↓, Mgat-1: ↓, SREBP1c: ↓, ChREBP: ↓, ACC: ↓, FAS: ↓	29
Adult spontaneous hypertensive rats	-	CQ (40 mg/kg/day, i.p., 21 days)	Body weight: ↓	COX enzymes: ↓, ROS: ↓, nitric oxide: ↑, MMP: ↓	67
Male C57BL mice	STZ (60 mg/kg, i.p.)	CQ (60 mg/kg/day, i.p., 14 days)	Body weight: ↔	LC3-II: ↑, p62: ↑, Beclin1: ↔, autophagic vacuoles: ↓	24

Abbreviations: ACC, acetyl-CoA carboxylase; Arg1, arginase 1; CD, cluster of differentiation; ChREBP, carbohydrate response element binding protein; COX, cyclooxygenase; CPT1α, carnitine palmitoyltransferase 1 alpha; CPT1β, carnitine palmitoyltransferase 1 beta; CQ, chloroquine; FAS; fatty acid synthase; HCQ, hydroxychloroquine; IL-1β, interleukin-1 beta; IL-6, interleukin-6; i.p., intraperitoneal; MCP-1, monocyte chemoattractant protein-1; Mgat-1, monoacylglycerol-O-acyltransferase; MMP, matrix metallopeptidase; PPAR-γ, peroxisome proliferator-activated receptor-gamma; ROS, reactive oxygen species; SREBP1c, sterol regulatory element-binding transcription factor 1; STZ, streptozotocin; TNF-α, tumour necrosis factor-alpha; ↑, increase/stimulate; ↓, decrease/inhibit; ↔, no change.

**Table 8:** The anti-obesity effects of CQ and HCQ in human studies

Study population	Treatment, dose and duration	Research outcomes	Mechanism of action	Reference
Healthy controls (n=34) and Q fever endocarditis patients (n=48) (mean age: 57 ± 15 years)	Doxycycline (100 mg, twice a day) + HCQ (600 mg daily)	Body weight: ↑	-	69

Abbreviations: HCQ, hydroxychloroquine; ↑, increase.
